# Enhancement of Anti-Staling Properties of Rice Bread Through Fermentation Rice Flour with Three Lactic Acid Bacteria

**DOI:** 10.3390/foods14152674

**Published:** 2025-07-29

**Authors:** Zhiqi Wang, Zhaosen Yuan, Xinlai Dou, Wanshan Yang, Huining Zhang, Yue Zhang, Fenglian Chen, Yanling Hao

**Affiliations:** 1 College of Food Engineering, Harbin University of Commerce, Harbin 150028, China; magiclynnia@163.com (Z.W.); 18665282812@163.com (Z.Y.); m13555016561@163.com (W.Y.); lemonzhn0923@163.com (H.Z.); 15046081883@163.com (Y.Z.); 2 Institute of Nutrition and Health, China Agricultural University, Beijing 100083, China; douxinlai2024@163.com

**Keywords:** rice bread, anti-staling, lactic acid bacteria, fermented rice flour

## Abstract

This study investigated the effects of *Lactococcus lactis* subsp. 1.2472 (L)-, *Streptococcus thermophilus* 1.2718 (S)-, and thermostable *Lactobacillus rhamnosus* HCUL 1.1901-1912 (T)-fermented rice flour with inoculum levels of 3–11% (*w*/*w*) on rice bread staling. Optimal staling resistance was achieved, as follows: 9% L-fermented rice bread (LRB), 7% T-fermented rice bread (TRB), and 5% S-fermented rice bread (SRB). Lactic acid bacteria-fermented rice flour significantly enhanced hydration properties. LF-NMR analysis revealed that T_21_ (strongly bound water) and T_22_ (weakly bound water) relaxation times decreased, while T_23_ (free water) increased with prolonged storage. Fermented-rice-flour groups had significantly more strongly bound water than the control group on 7 d. The optimized formulations exhibited exceptional volumetric stability with specific volume change rates of 17.63% (LRB), 17.60% (TRB), and 19.58% (SRB), coupled with maximal porosities of 10.34%, 9.05%, and 9.41%, respectively. This study provides a theoretical foundation for improving rice bread’s anti-staling properties.

## 1. Introduction

Rice is a global staple predominantly consumed as cooked grains or processed into flour-based products (e.g., rice cakes, cookies, and bread) [1,2,3,4]. However, rice-flour-based products usually face staling challenges due to their high starch content (70–80% dry weight) [5,6]. This polysaccharide undergoes rapid recrystallization during post-processing cooling and storage, particularly problematic in rice bread where staling manifests through the following dual mechanisms: starch retrogradation and moisture redistribution [7].

Starch retrogradation—the time-dependent reorganization of gelatinized starch molecules via intra- and intermolecular double-helix formation [8]—constitutes the primary aging pathway in starch matrices. Contemporary anti-staling strategies employ the following three approaches: (1) modifications of raw material powder, (2) hydrocolloid supplementation, and (3) functional additives. Alginate incorporation created biphasic systems where a continuous aqueous phase encapsulated dispersed starch granules, enhancing product softness through water mobility modulation [9]. Maillard reaction products (aldehydic acids and heterocyclic acids/ketones) inhibited moisture evaporation and starch degradation and stabilized gluten networks [10]. Contrastingly, microwave-modified rice flour exhibited increased amylose content and crystallinity, delaying staling [11].

Fermentation technologies leverage microbial metabolism to simultaneously inhibit starch retrogradation and improve moisture retention while enhancing flavor profiles [12,13]. Chickpea-enriched sourdough fermentation, for instance, increased gluten-free bread specific volume while reducing crumb hardness and retrograded amylopectin enthalpy during storage [14].

Notably, the application of lactic acid bacteria (LAB)-fermented rice flour in bread systems remains underexplored. Research about fermented rice flour have primarily focused on reducing protein removal rates and increasing gamma-aminobutyric acid content. In contrast, this study emphasizes the effect of lactic acid bacteria fermentation on bread texture, particularly the impact of inoculum size on staling kinetics [15,16]. Therefore, this study aims to investigate how three LAB strains fermenting rice flour influence the anti-staling properties of rice bread and dose–response relationships between fermentation load (3–11% *w*/*w*) and product stability. The findings provide a theoretical basis for enhancing rice bread quality during storage.

## 2. Materials and Methods

### 2.1. Materials

*Lactococcus lactis* subsp. (strain: 1.2472) (L) and *Streptococcus thermophilus* (strain:1.2718) (S) were purchased from China General Microbiological Culture Collection Center (CGMCC). Thermostability *lactobacillus rhamnosus* HCUL 1.1901-1912 (T) were preserved in the Laboratory of the College of Food Engineering. The polished round-grained rice (protein 5.3%, lipid 0.8%, and carbohydrate 77.6%) was selected from Yanbian, China. Hydroxypropyl methylcellulose (HPMC) was purchased from Qianwei Food Technology Co., Ltd. (Shanghai, China). Wheat flour (protein 12.2%, lipid 1.6%, and carbohydrate 72.3%) was supplied by Yihai Kerry Arawana Holdings Co., Ltd. (Shanghai, China). Milk powder was obtained from Guangming Songhe Dairy Co., Ltd. (Harbin, China). Butter was selected from Deyang Food Co., Ltd. (Shanghai, China). Wheat gluten was purchased from Zhengzhou Chenyang Chemical Co., Ltd. (Zhengzhou, China). Sucrose, yeast, egg, and salt were purchased from local markets. Anhydrous ethanol, sodium chloride, and MRS broth medium were all of analytical grade.

### 2.2. Preparation of Fermented Rice Flour and Rice Bread (RB)

#### 2.2.1. Microorganisms Culture

Three probiotic strains (L, S, and T) were stored at −80 °C in 25% glycerol. Prior to use, each strain of microorganisms was inoculated into separate sterile MRS broth media, mixed thoroughly, and incubated statically at 37 °C for 24 h. The activation process was repeated twice continuously until the viable cell count reached approximately 10^8^ CFU/mL. Then, they were used for the subsequent analyses.

#### 2.2.2. Preparation of Fermented Rice Flour

Rice flour (RF) was prepared as follows: rice was mixed with distilled water at 1:3 (*w*/*v*) ratio in a 1000 mL Erlenmeyer flask. After standing for 2 h, the mixture was wet-milled, followed by centrifugation at 3000 rpm for 10 min. After discarding the supernatant, the precipitate was dried in a vacuum oven at 40 °C. The dried sample was subsequently sieved through an 80-mesh sieve to obtain rice flour.

The LAB-fermented RF was prepared as follows: The cleaned rice was transferred to 1 L Erlenmeyer flasks and homogenized with sterile distilled water at a 1:3 (*w*/*v*) ratio. For each bacterial strain, five inoculation levels (3%, 5%, 7%, 9%, and 11%, *v*/*w*) were evaluated for their impact on the staling properties of fermented rice bread. Each treatment was performed in triplicate. A predetermined volume of Strain L suspension was aseptically introduced into the rice–water mixture, vortexed to ensure homogeneity, and then incubated statically at 37 °C for 24 h. Post-fermentation, the L-fermented rice slurry underwent wet grinding and centrifugation (3000 rpm, 10 min) for supernatant removal. The resulting pellet was vacuum-dried at 40 °C and, subsequently, passed through an 80-mesh sieve to obtain L-fermented rice flour (LRF). T-fermented rice flour (TRF) and S-fermented rice flour (SRF) were prepared using the same method.

### 2.3. Production Process of Fermented Rice Bread 

Control group RB formulation comprised 40% RF, 60% wheat flour, 1.5% yeast, 18.0% butter, 18.0% sucrose, 8% egg, 0.6% salt, 1.5% HPMC, 3%wheat gluten, and 47.0% water (all percentages by weight relative to total flour basis). In the experimental group, 40% of RF was replaced with 40% LAB-fermented RF. All ingredients were mixed in a standard mixer (CS-B7, Changsheng Electrical Equipment Co., Ltd., Guangzhou, China) at 350 rpm for 5 min to form a dough. The dough was transferred to a baking tray subjected to primary fermentation at 37 °C with 75% relative humidity for 120 min. For baking, the oven was initially set to 170 °C (top heat) and 140 °C (bottom heat). The dough was baked at these temperatures for 7 min. Upon observing substantial volume expansion, the oven temperatures were adjusted to 160 °C (both top and bottom heat) for an additional 5 min. The bread was removed from the oven when its surface turned light yellow, indicating optimal browning. The prepared rice bread was stored unpackaged in an environment at 25 °C with 40% humidity for subsequent various tests.

### 2.4. RB Crumbs Moisture

Moisture content was determined for samples in different rice bread according to the AACC Official Method 44-15.02 [17].

### 2.5. Low-Field NMR (LF-NMR)

LF-NMR measurements of the doughs were performed using an NMR analyzer (NMI20-015 V-I, Niumag Co., Ltd., Suzhou, China) to determine the spin-spin relaxation time T2 of the bread at a frequency of 0.1 MHz and operating at 32 ± 0.1 °C. The central portion of each bread sample was cut into a cylindrical shape (1 cm diameter and 2–3 cm height). Samples were wrapped in plastic film to prevent moisture loss and then placed into a 15 mm NMR sample tube. The relaxation delay for repeatable sampling was set to 1000 ms.

### 2.6. Specific Loaf Volume (SLV)

After baking, bread samples were cooled to room temperature prior to quality and volume measurements. Mass was measured using an electronic balance (TD20002A, Shanghai Lichen Instrument Technology Co., Ltd. Shanghai, China), and volume was determined via a rapeseed displacement method. SLV was calculated as the ratio of volume to mass, expressed in units of mL/g [18].

### 2.7. Determination of Porosity

Five bread loaves from the same batch were sliced into uniform slices. Cross-sectional texture images of the slices were acquired at 600 dpi using an image scanner, and a central 3 cm × 3 cm region was cropped from each image. The cropped images were converted to 8-bit grayscale and processed with ImageJ software (v1.51j8; National Institutes of Health, Bethesda, MD, USA). Pixel values were calibrated to physical length units using a reference standard of known dimensions. Images were binarized—pores in black and crumb matrix in white—to determine pore density (number of pores per cm^2^) and percentage pore area [19].

### 2.8. Determination of Sensory Evaluation

Bread samples were first sectioned into evenly sized pieces, which were then split widthwise into halves to prepare test samples. The sensory evaluation panel comprised 40 participants (graduate school students from the College of Food Engineering, Harbin University of Commerce), including 20 males and 20 females, aged between 22 and 27. All participants received systematic training and achieved excellent performance in sensory evaluation courses. Panelists evaluated the bread according to their acceptance of six attributes, as follows: color, appearance, tactile sensation, taste, internal structure, and crumb color. Scoring criteria allocated 15 points maximum for color, appearance, internal structure, and crumb color; tactile sensation and taste each carried a 20-point maximum. Following score compilation, mean values were calculated, with the total assessment score capped at 100 points. Sensory evaluation methods refer to ASTM E2610-18 [20].

### 2.9. Statistical Analysis

Statistical analyses were performed using SPSS 23.0, while graphical plotting and data visualization were conducted with Origin 2022. Following a one-way analysis of variance (ANOVA), Tukey’s honest significant difference (HSD) post hoc test was applied to determine significant differences between sample groups. Statistical significance was defined as *p* < 0.05, and all experimental measurements were performed in triplicate to ensure reproducibility.

## 3. Results and Discussions

### 3.1. Moisture Content

**Figure 1:**
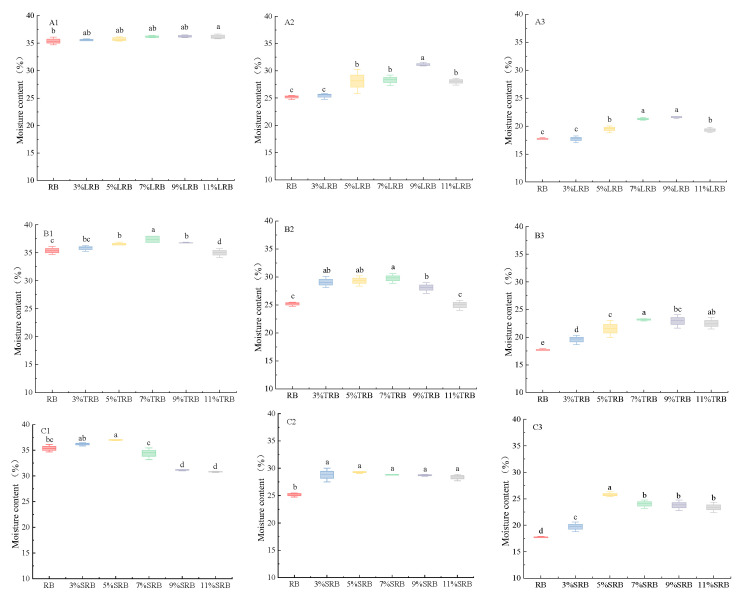
The changes in the moisture content of rice bread fermented by different LAB over different storage times: (**A**–**C**) LRB, TRB, and SRB; (**1**–**3**) storage for 1d, 4d, and 7d. LRB, TRB, and SRB represent *Lactococcus-lactis*-fermented rice bread, thermostable *Lactobacillus-rhamnosus*-fermented rice bread, and *Streptococcus-thermophilus*-fermented rice bread. Different letters in the same row indicate significant differences (*p* < 0.05).

As shown in Figure 1, all rice bread samples demonstrated progressive moisture loss during prolonged storage. This phenomenon primarily arose from two distinct mechanisms. First, low ambient humidity during storage accelerated surface moisture evaporation, creating a vapor pressure gradient that drove internal moisture migration toward the bread surface. This moisture transferred ultimately reduced crumb moisture content and increased product dryness [20]. Second, starch retrogradation occurred through recrystallization of gelatinized starch molecules, a physicochemical process associated with starch aging [21].

A positive correlation was observed between the bacterial inoculum volume and moisture retention in the LRB, as evidenced by the consistent trends in Figure 1(A1–A3). The 9% LRB exhibited maximum moisture retention, with recorded values of 36.31%, 31.05%, and 21.67%, as shown in Figure 1(A1–A3), respectively. As demonstrated in Figure 1(B1–C3), moisture preservation in rice bread is dependent on inoculum levels. At optimal volume, the 7% TRB and 5% SRB maintained elevated moisture retention throughout storage. The 7% TRB displayed moisture contents of 37.42%, 29.81%, and 23.25% (Figure 1(B1–B3)), while the 5% SRB exhibited values of 37.05%, 28.85%, and 25.74% (Figure 1(C1–C3)). The enhanced moisture retention was primarily attributed to enzymatic activity during rice flour fermentation, amylases and proteases catalyzed the hydrolysis of starch granules and globular proteins into low-molecular-weight saccharides, oligopeptides, and free amino acids [14]. These biochemical modifications improved the structural integrity of rice bread [22], thereby enhancing both gas entrapment efficiency and water-binding capacity within the dough system, ultimately increasing final product moisture content. On the other hand, amino acids and reducing sugars generated via enzymatic degradation participated in Maillard reaction pathways, accelerating crust-browning kinetics while reducing optimal baking duration and mitigating moisture depletion during thermal processing. Notably, the moisture retention of the 11% LRB decreased, which may be attributed to the lactic acid produced by the LAB during fermentation, resulting in a reduction in pH [23]. An excessive inoculum concentration caused the fermented rice flour mixture to be in an overly acidic state. This acidic shift compromised the structural coherence of the protein network, significantly diminishing the dough’s hydration capacity.

### 3.2. Water Migration

**Figure 2:**
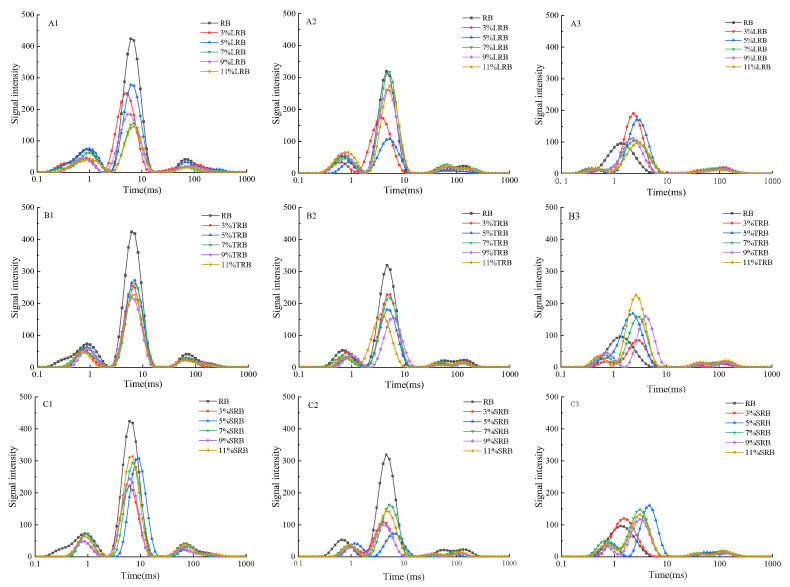
T2 inversion plots of the water transverse relaxation time for rice breads fermented with different LAB at various storage times: (**A**–**C**) represent LRB, TRB, and SRB; (**1**–**3**) represent storage 1d, 4d, and 7d. LAB represent the lactic acid bacteria. LRB, TRB, and SRB represent *Lactococcus-lactis*-fermented rice bread, thermostable *Lactobacillus-rhamnosus*-fermented rice bread, and *Streptococcus-thermophilus*-fermented rice bread.

Figure 2 revealed three characteristic peaks in each spectrum, corresponding to distinct moisture states within the samples. The T_21_ component represents tightly bound water molecules that interacting with starch and gluten proteins through hydrogen bonds and dipole interactions. T_22_ signifies loosely bound water displaying intermediate mobility, confined within the three-dimensional macromolecular networks formed by protein and starch matrices. T_23_ corresponds to bulk free water [24]. During storage, progressive attenuation of all peak intensities was observed, attributable to starch recrystallization and retrogradation processes within the bread matrix. This phenomenon resulted in significant moisture redistribution, causing progressive loss of both bound and free water fractions at varying rates.

Comparative analysis of Table 1, Table 2 and Table 3 demonstrated consistent trends in LAB-fermented rice bread that the T_21_ and T_22_ relaxation times decreased systematically with extended storage, whereas the T_23_ values increased with prolonged storage duration. These patterns suggested enhanced binding interactions between water molecules and substrate matrices (strongly/weakly bound fractions) alongside elevated mobility of unbound water phases. Moisture redistribution of LRB was further evidenced by progressive A_21_ reduction concurrent with A_22_ and A_23_ increases, reflecting the migration of structurally bound water into semi-bound and free stages during storage [25]. Moisture redistribution of TRB and SRB is not very obvious.

Following 7-day storage, RB exhibited significantly reduced tightly bound water content and elevated free water levels relative to other LAB-fermented flour rice bread. Formulations containing 9% LRB, 7% TRB, and 5% SRB achieved optimal moisture retention, demonstrating minimal T_21_/T_22_ relaxation times and A_22_ amplitudes, coupled with maximal A_21_ amplitudes. These parameters reflect restricted water mobility coupled with enhanced binding interactions between water molecules and other components within the bread matrix. This effectively inhibited moisture migration while retarding starch retrogradation. These findings are consistent with previous measurements of moisture content.

### 3.3. Porosity

**Figure 3:**
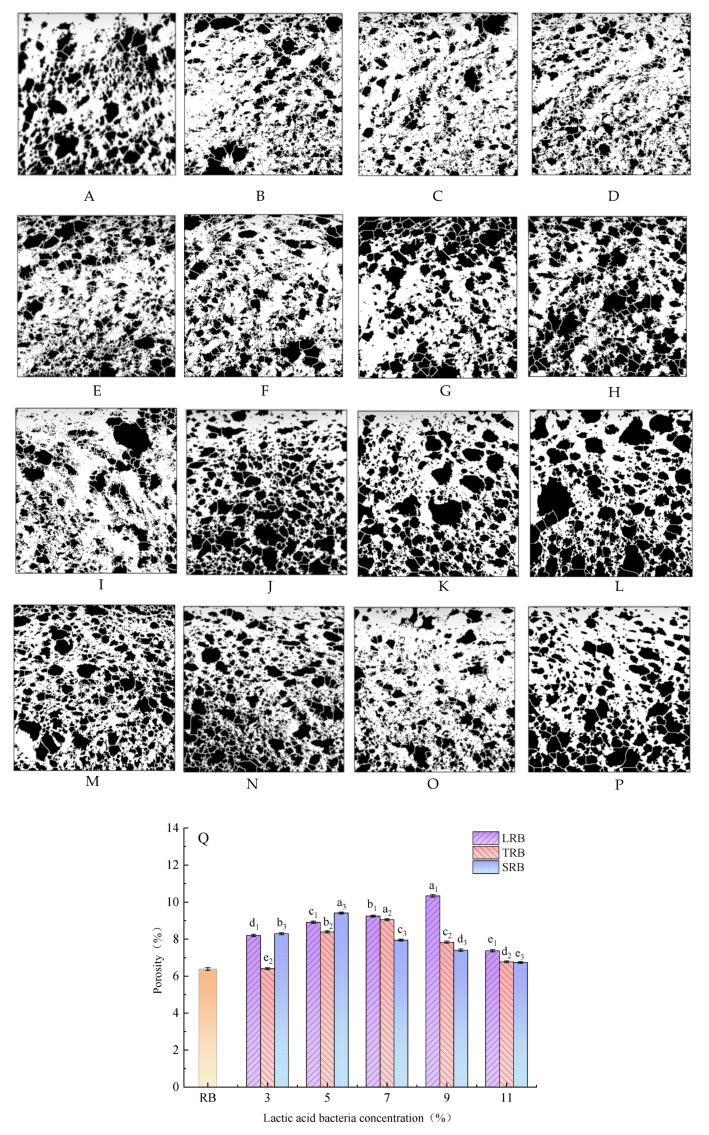
Porosity (**A**–**P**) and porosity changes (**Q**) of bread supplemented with different bacterial strains at various addition dosages: (**A**) porosity of RB; (**B**–**F**) porosity of LRB added at 3%, 5%, 7%, 9%, and 11%; (**G**–**K**) porosity of TRB added at 3%, 5%, 7%, 9%, and 11%; (**L**–**P**) porosity of SRB added at 3%, 5%, 7%, 9%, and 11%. LRB, TRB, and SRB represent *Lactococcus-lactis*-fermented rice bread, thermostable *Lactobacillus-rhamnosus*-fermented rice bread, and *Streptococcus-thermophilus*-fermented rice bread. Different letters in the same row indicate significant differences (*p* < 0.05).

Crumb texture is fundamentally determined by cellular architecture compactness, a key structural parameter governing specific volume and sensory characteristics. Elevated porosity typically correlates with enhanced baking quality, defined as the void volume fraction within bread crumb (total pore volume per unit bread volume) [26]. Crumb structures containing numerous large pores demonstrate reduced porosity values, whereas uniform pore size distributions yield higher porosity indices [27]. As evidenced by Figure 3, RB displayed significantly lower porosity (6.39%) compared to LAB-fermented rice-flour bread. The LRB attained peak porosity (10.34%) at 9% inoculum, TRB achieved 9.05% at a 7% addition, while SRB reached 9.41% at a 5% supplementation.

Porosity exhibited progressive elevation with increasing inoculum concentration up to critical thresholds. During fermentation, carbon dioxide became entrapped within the developing gluten–starch matrix, creating stabilized gas cells that impart characteristic crumb loftiness. This phenomenon principally originated from LAB-mediated starch hydrolysis, generating fermentable sugars that accelerated yeast metabolism, thereby shortening fermentation duration while enhancing CO_2_ generation rates. Beyond optimal inoculum levels (9% LRB, 7% TRB, and 5% SRB), porosity displayed a reduction despite continued CO_2_ production. This reversal mainly occurred because CO_2_ pressure exceeded the critical value that the bread structure could withstand, causing pore wall rupture and overall structural collapse [28]. These findings were corroborated by previous studies, demonstrating enhanced pore stability in LAB-fermented, steamed multigrain bread.

### 3.4. SLV

**Figure 4:**
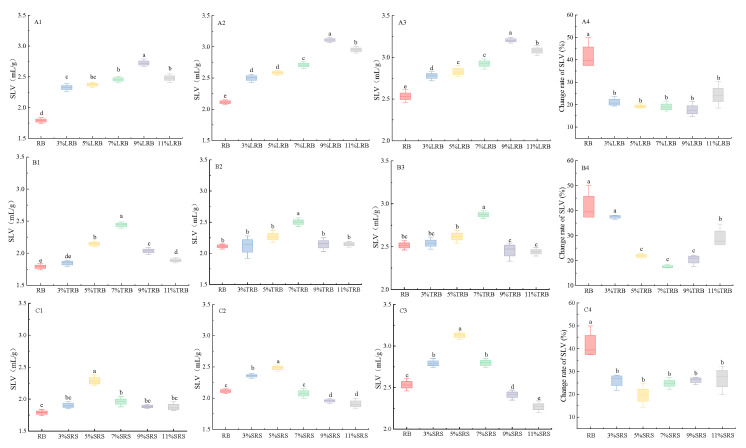
The specific volume changes in rice bread fermented by different LAB over various storage times: (**A**–**C**) LRB, TRB, and SRB; (**1**–**4**) storage for 1d, 4d, and 7d and the specific volume change rate. LRB, TRB, and SRB represent *Lactococcus-lactis*-fermented rice bread, thermostable *Lactobacillus-rhamnosus*-fermented rice bread, and *Streptococcus-thermophilus*-fermented rice bread. Different letters in the same row indicate significant differences (*p* < 0.05).

As shown in Figure 4, LAB fermentation significantly modulated the specific volume of rice bread. The specific volumes of breads fermented with three LAB strains exhibited biphasic responses to the inoculum concentration, peaking at 9%, 7%, and 5%, respectively, with a 63–73% volume enhancement. This optimal dosage effect arises from LAB-derived exopolysaccharides functioning as natural hydrocolloids that reinforce protein matrix cross-linking, thereby improving gas retention capacity [29]. Moreover, moderate LAB concentrations (6%) synergistically enhanced yeast fermentation kinetics by pH-mediated α-amylase activation accelerating CO_2_ production [30]. Therefore, the specific volume of the bread increased. However, supra optimal inoculum induced over-acidification, triggering arabinoxylan hydrolysis, and structural protein degradation, ultimately compromising bread architecture [31,32]. These findings align with Ketabi et al. [33], who reported that exopolysaccharides mediated specific volume improvements in fermented cereal systems through enhanced dough viscoelasticity.

SLV was calculated as [(V_1_ − V_7_)/V_1_] × 100%, where V_1_ and V_7_ represent specific volumes at 1-day and 7-day storage, respectively. As shown in Figure 4, SLV displayed parabolic trends with the LAB inoculum concentration (0–11% *v*/*v*), reaching minimal values (17.63 ± 0.85%) at a 9% LRB dosage (Figure 4(A4)). This was primarily due to the fact that the volume of bread remained constant during storage, while only the mass underwent change. Therefore the rate of specific volume change was minimal, as was the change in mass, which suggests that the moisture content of the bread also exhibited little variation. Similar patterns emerged for the TRB and SRB strains (Figure 4( B1–C3)). The TRB demonstrated peak anti-aging performance at 7% inoculum (SLV: 17.60 ± 1.02%), while the SRB showed optimal results at 5% (SLV: 19.58 ± 1.15%), demonstrating their enhanced anti-aging capacity. This aligns with results from the water content and migration distribution experiments.

### 3.5. Sensory Evaluation

**Figure 5:**
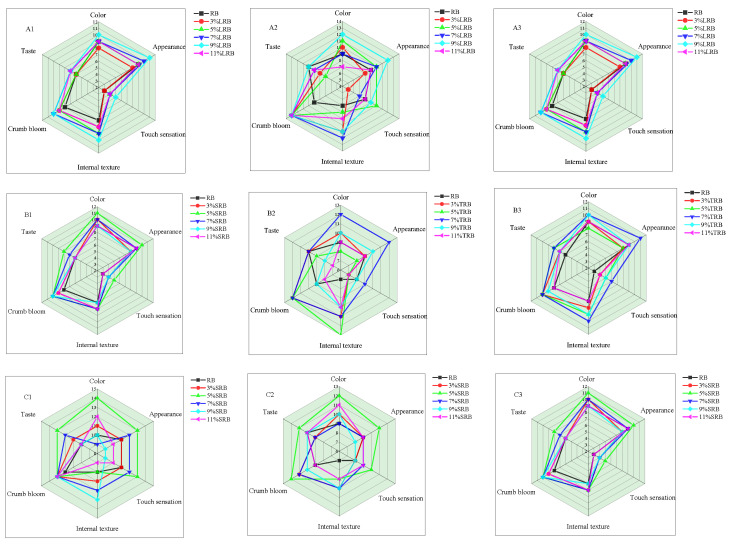
Sensory evaluation of rice bread fermented by different LAB over various storage times: (**A**–**C**) LRB, TRB, and SRB; (**1**–**3**) storage for 1d, 4d, and 7d. LRB, TRB, and SRB represent the *Lactococcus-lactis*-fermented rice bread, thermostable *Lactobacillus-rhamnosus*-fermented rice bread, and *Streptococcus-thermophilus*-fermented rice bread.

The comprehensive sensory evaluation protocol encompassed exterior attributes (color, appearance, and touch sensation) and intrinsic quality parameters (taste, internal structure, and crumb color). As depicted in Figure 5, internal structure was the most significantly affected sensory evaluation indicator, with its scores decreasing systematically as storage time increased (1, 4, and 7 days). This phenomenon is associated with starch retrogradation—which causes textural firming, flavor degradation, and structural collapse. Therefore, if the structural stability of rice bread can be further enhanced and structural damage during storage can be improved, its sensory quality, especially touch sensation and taste, is expected to be more significantly optimized. Figure 5(A1–A3) indicate that the LRB with 9% inoculum achieved the highest sensory scores across all storage durations. This superiority can be ascribed to the acidic environment generated by enhanced inoculation of LAB. Their metabolites inhibited water migration between proteins and starch during storage thereby improving water-holding capacity. The incorporation of LAB-fermented rice flour optimized the dough network structure by enhancing gas retention and improving visual appeal while rendering its internal texture more delicate with a loose and porous state [34]. Compared to RB, the LAB-fermented rice-flour bread exhibited a more pronounced rice aroma due to the production of various flavor compounds resulting from LAB-mediated hydrolysis of proteins along with carbohydrate and fat metabolism [35]. Furthermore, the intrinsic fragrance from rice flour alleviated the characteristic sourness associated with fermented bread, significantly enhancing overall palatability. Dan et al. [36] reported that upon LAB-fermented sourdough addition, green wheat-kernel-flour bread displayed superior flavor and texture. The results presented in Figure 5(B1–B3,C1–C3) demonstrate that rice bread prepared with 7% TRB and 5% SRB bacterial inoculations exhibited optimal sensory performance during storage. This was accompanied by enhanced anti-aging properties.

## 4. Conclusions

This study investigated the anti-staling effects of rice flour fermented by LAB (strains L, S, and T) on rice bread during 1–7 days of storage, with a focus on changes in baking quality. Compared with the non-fermented control group, LAB fermentation significantly improved the sensory and textural properties of bread by effectively inhibiting starch retrogradation. Among the formulations, the optimal water retention capacity was observed in those supplemented with 9% LRB, 7% TRB, and 5%SRB, with moisture contents of 21.67%, 23.25%, and 25.74% after 7 days of storage, respectively. Additionally, metabolites produced by LAB enhanced the integrity of the protein matrix during storage, inhibiting the conversion of bound water to free water and thereby further improving water retention.

Analysis integrating moisture content, LF-NMR, pore architecture, and specific volume indicated that LAB fermentation enhanced the structural coherence of rice bread. Specifically, this was manifested as an increased proportion of T_21_, a decreased proportion of T_23_, and suppressed water loss between starch–protein complexes. Concurrently, rice bread exhibited homogenized pore distribution, a 63–73% increase in specific volume, and the lowest specific volume change rates during storage (17.63%, 17.60%, and 19.58% for 9% LRB, 7% TRB, and 5% SRB, respectively). These factors collectively delayed rice bread staling.

However, the rice bread formulation in this study contained 60% wheat flour, and we have not yet systematically analyzed the specific mechanism by which this proportion affects the structure of rice bread. Meanwhile, the mechanism of action of LAB-fermented rice flour requires further investigation. Future studies could isolate and purify LAB metabolites and conduct analysis at the dough level to clarify their regulatory effects on starch retrogradation and water binding.

## Figures and Tables

**Table 1:** LRB moisture changes over different storage times.

Storage Day	Sample	T2/ms			Relative Peak Area/%		
		T_21_/ms	T_22_/ms	T_23_/ms	A_21_/%	A_22_/%	A_23_/%
1d	RB	0.87 ± 0.00 ^bc^	6.14 ± 0.00 ^b^	65.79 ± 7.18 ^a^	16.30 ± 1.97 ^c^	75.84 ± 2.62 ^a^	7.87 ± 0.65 ^a^
	3%LRB	0.87 ± 0.00 ^bc^	5.34 ± 0.00 ^c^	71.51 ± 37.65 ^c^	18.93 ± 0.76 ^c^	73.78 ± 1.76 ^a^	7.29 ± 2.32 ^a^
	5%LRB	0.96 ± 0.08 ^ab^	6.14 ± 0.00 ^b^	69.08 ± 5.69 ^a^	25.06 ± 1.25 ^b^	67.79 ± 1.14 ^b^	7.14 ± 0.13 ^a^
	7%LRB	1.00 ± 0.00 ^a^	7.05 ± 0.00 ^a^	65.79 ± 0.00 ^a^	29.33 ± 2.81 ^a^	64.07 ± 2.40 ^c^	6.60 ± 0.41 ^a^
	9%LRB	0.83 ± 0.07 ^c^	5.60 ± 0.46 ^c^	63.36 ± 10.64 ^a^	29.91 ± 1.19 ^c^	62.55 ± 0.68 ^a^	7.54 ± 1.87 ^a^
	11%LRB	1.00 ± 0.00 ^a^	7.05 ± 0.00 ^a^	72.36 ± 5.69 ^a^	26.20 ± 1.64 ^b^	65.90 ± 1.56 ^bc^	7.89 ± 0.13 ^a^
4d	RB	0.69 ± 0.06 ^cd^	4.64 ± 0.00 ^b^	57.22 ± 0.00 ^b^	13.40 ± 0.36 ^c^	80.79 ± 0.22 ^a^	5.81 ± 0.43 ^a^
	3%LRB	0.55 ± 0.11 ^d^	3.51 ± 0.00 ^c^	113.34 ± 32.65 ^a^	12.50 ± 1.55 ^c^	79.67 ± 2.16 ^b^	7.82 ± 3.52 ^a^
	5%LRB	0.96 ± 0.08 ^a^	5.34 ± 0.00 ^a^	72.85 ± 12.23 ^b^	13.30 ± 1.39 ^a^	78.10 ± 1.59 ^c^	8.60 ± 0.32 ^a^
	7%LRB	0.87 ± 0.12 ^ab^	5.34 ± 0.00 ^a^	62.94 ± 4.95 ^b^	14.09 ± 0.34 ^c^	79.08 ± 0.40 ^c^	6.83 ± 0.73 ^a^
	9%LRB	0.76 ± 0.11 ^bc^	5.11 ± 0.40 ^a^	60.08 ± 4.95 ^b^	18.48 ± 1.18 ^b^	74.64 ± 0.82 ^d^	6.88 ± 1.96 ^a^
	11%LRB	0.83 ± 0.07 ^abc^	5.34 ± 0.00 ^a^	62.94 ± 4.95 ^b^	17.73 ± 0.30 ^a^	75.70 ± 0.55 ^dc^	6.57 ± 0.38 ^a^
7d	RB	0.52 ± 0.00 ^b^	3.33 ± 0.00 ^a^	100.00 ± 0.00 ^b^	0.06 ± 0.02 ^d^	85.72 ± 0.05 a	14.22 ± 0.64 ^a^
	3%LRB	0.48 ± 0.04 ^b^	2.31 ± 0.00 ^c^	111.38 ± 22.82 ^a^	2.54 ± 2.15 ^c^	87.11 ± 3.06 ^a^	10.35 ± 4.40 ^ab^
	5%LRB	0.42 ± 0.07 ^b^	2.79 ± 0.23 ^b^	121.46 ± 18.59 ^a^	6.97 ± 0.43 ^b^	83.57 ± 2.39 ^ab^	9.46 ± 2.01 ^b^
	7%LRB	0.48 ± 0.08 ^b^	2.66 ± 0.00 ^b^	100.65 ± 14.01 ^a^	7.41 ± 2.78 ^ab^	79.94 ± 1.27 ^bc^	12.64 ± 1.60 ^ab^
	9%LRB	0.43 ± 0.12 ^b^	2.21 ± 0.17 ^c^	114.98 ± 0.00 ^a^	9.50 ± 1.83 ^b^	77.02 ± 1.80 ^bc^	13.48 ± 0.04 ^ab^
	11%LRB	0.45 ± 0.04 ^b^	2.66 ± 0.00 ^b^	96.31 ± 16.17 ^a^	8.37 ± 2.00 ^a^	79.94 ± 2.35 ^c^	11.69 ± 2.05 ^ab^

Different letters in the same row indicate significant differences (*p* < 0.05). Data represent the mean (±SD) from three independent replicate experiments. LRB, represents the *Lactococcus-lactis*-fermented rice bread.

**Table 2:** TRB moisture changes over different storage times.

Storage Day	Sample	T2/ms			Relative Peak Area/%		
		T_21_/ms	T_22_/ms	T_23_/ms	A_21_/%	A_22_/%	A_23_/%
1d	RB	0.87 ± 0.00 ^ab^	6.14 ± 0.00 ^a^	65.79 ± 0.00 ^b^	16.30 ± 1.97 ^c^	75.84 ± 2.62 ^a^	7.87 ± 0.65 ^a^
	3%TRB	0.76 ± 0.00 ^c^	6.14 ± 0.00 ^a^	65.79 ± 0.00 ^b^	16.55 ± 0.07 ^b^	75.27 ± 0.16 ^a^	8.18 ± 0.17 ^c^
	5%TRB	0.91 ± 0.08 ^a^	7.05 ± 0.00 ^a^	65.79 ± 0.00 ^b^	17.85 ± 0.16 ^a^	73.62 ± 0.74 ^cd^	8.53 ± 0.58 ^bc^
	7%TRB	0.79 ± 0.07 ^bc^	7.05 ± 0.00 ^a^	75.65 ± 0.00 ^a^	16.57 ± 0.35 ^b^	74.31 ± 0.26 ^bc^	9.12 ± 0.58 ^b^
	9%TRB	0.87 ± 0.00 ^ab^	6.14 ± 0.00 ^a^	62.94 ± 4.95 ^b^	16.64 ± 0.18 ^b^	72.92 ± 0.24 ^d^	10.44 ± 0.06 ^a^
	11%TRB	0.76 ± 0.00 ^c^	7.05 ± 0.20 ^a^	69.08 ± 5.69 ^ab^	15.71 ± 0.58 ^c^	75.01 ± 0.57 ^ab^	9.28 ± 0.59 ^b^
4d	RB	0.69 ± 0.06 ^c^	4.64 ± 0.00 ^a^	57.22 ± 0.00 ^b^	13.40 ± 0.36 ^c^	80.79 ± 0.22 ^a^	5.81 ± 0.43 ^a^
	3%TRB	0.87 ± 0.00 ^ab^	5.34 ± 0.00 ^a^	47.61 ± 3.74 ^b^	14.59 ± 0.40 ^ab^	80.44 ± 0.45 ^b^	4.97 ± 0.26 ^a^
	5%TRB	0.83 ± 0.07 ^abc^	4.64 ± 0.00 ^a^	45.45 ± 3.74 ^b^	14.06 ± 0.59 ^b^	81.20 ± 0.47 ^ab^	4.74 ± 0.12 ^a^
	7%TRB	0.76 ± 0.11 ^bc^	5.34 ± 0.15 ^a^	49.77 ± 0.00 ^b^	12.07 ± 0.58 ^c^	82.79 ± 0.20 ^a^	5.15 ± 0.41 ^a^
	9%TRB	0.96 ± 0.08 ^a^	6.14 ± 0.00 ^a^	71.51 ± 37.65 ^b^	15.34 ± 0.40 ^a^	78.30 ± 1.64 ^c^	6.36 ± 2.03 ^a^
	11%TRB	0.76 ± 0.00 ^bc^	3.51 ± 0.00 ^a^	120.72 ± 9.94 ^a^	11.79 ± 0.22 ^c^	82.32 ± 0.69 ^a^	5.89 ± 0.61 ^a^
7d	RB	0.52 ± 0.00 ^b^	3.33 ± 0.00 ^a^	100.00 ± 0.00 ^b^	0.06 ± 0.02 ^d^	85.72 ± 0.05 a	14.22 ± 0.64 ^a^
	3%TRB	0.76 ± 0.00 ^b^	2.92 ± 0.23 ^c^	120.72 ± 9.94 ^a^	15.49 ± 1.17 ^b^	77.21 ± 1.28 ^b^	7.31 ± 0.64 ^a^
	5%TRB	0.63 ± 0.05 ^c^	2.92 ± 0.23 ^c^	145.39 ± 11.43 ^a^	8.99 ± 0.56 ^c^	83.14 ± 0.02 ^ab^	7.87 ± 0.57 ^a^
	7%TRB	0.53 ± 0.08 ^d^	2.31 ± 0.00 ^d^	138.79 ± 11.43 ^a^	20.07 ± 1.31 ^b^	73.69 ± 1.25 ^b^	6.25 ± 0.11 ^bc^
	9%TRB	0.72 ± 0.06 ^b^	4.04 ± 0.00 ^b^	107.20 ± 43.28 ^a^	19.57 ± 0.80 ^a^	74.55 ± 0.86 ^c^	5.88 ± 0.09 ^c^
	11%TRB	0.52 ± 0.04 ^d^	2.66 ± 0.00 ^c^	145.39 ± 11.43 ^a^	10.32 ± 0.57 ^c^	83.11 ± 0.56 ^ab^	6.57 ± 0.14 ^b^

Different letters in the same row indicate significant differences (*p* < 0.05). Data represent the mean (±SD) from three independent replicate experiments. TRB represents the thermostable *Lactobacillus-rhamnosus*-fermented rice bread.

**Table 3:** SRB moisture changes over different storage times.

Storage Day	Sample	T2 /ms			Relative Peak Area/%		
		T_21_/ms	T_22_/ms	T_23_/ms	A_21_/%	A_22_/%	A_23_/%
1d	RB	0.87 ± 0.00 ^ab^	6.14 ± 0.00 ^c^	65.79 ± 0.00 ^b^	16.30 ± 1.97 ^c^	75.84 ± 2.62 ^a^	7.87 ± 0.65 ^a^
	3%SRB	0.87 ± 0.00 ^ab^	6.14 ± 0.00 ^c^	60.45 ± 9.25 ^b^	15.39 ± 0.26 ^a^	75.96 ± 1.15 ^a^	8.65 ± 1.38 ^a^
	5%SRB	0.96 ± 0.08 ^a^	9.33 ± 0.00 ^a^	86.97 ± 0.00 ^a^	15.51 ± 0.21 ^a^	76.47 ± 0.14 ^a^	8.02 ± 0.09 ^a^
	7%SRB	0.91 ± 0.08 ^ab^	7.05 ± 0.00 ^b^	72.36 ± 5.69 ^b^	15.32 ± 0.17 ^a^	75.80 ± 0.62 ^a^	8.88 ± 0.45 ^a^
	9%SRB	0.79 ± 0.07 ^b^	6.14 ± 0.00 ^c^	65.79 ± 0.00 ^b^	14.67 ± 0.31 ^b^	76.73 ± 0.42 ^a^	8.59 ± 0.19 ^a^
	11%SRB	0.83 ± 0.07 ^ab^	6.75 ± 0.53 ^b^	69.08 ± 5.69 ^b^	14.64 ± 0.39 ^b^	76.31 ± 0.43 ^a^	9.10 ± 0.29 ^a^
4d	RB	0.69 ± 0.06 ^c^	4.64 ± 0.00 ^c^	57.22 ± 0.00 ^c^	13.40 ± 0.36 ^c^	80.79 ± 0.22 ^a^	5.81 ± 0.43 ^bc^
	3%SRB	1.00 ± 0.00 ^b^	4.04 ± 0.00 ^c^	115.72 ± 6.11 ^b^	17.21 ± 1.26 ^b^	77.62 ± 1.55 ^ab^	5.16 ± 0.29 ^c^
	5%SRB	0.911 ± 0.10 ^a^	3.94 ± 0.53 ^a^	86.97 ± 0.00 ^b^	31.93 ± 0.86 ^a^	61.65 ± 0.92 ^c^	6.42 ± 0.12 ^ab^
	7%SRB	0.96 ± 0.08 ^b^	5.34 ± 0.00 ^b^	121.46 ± 8.59 ^a^	16.19 ± 1.30 ^b^	78.41 ± 0.26 ^a^	5.40 ± 1.04 ^bc^
	9%SRB	1.00 ± 0.00 ^b^	4.24 ± 0.35 ^c^	100.65 ± 4.01 ^b^	17.58 ± 1.06 ^b^	75.71 ± 0.68 ^b^	6.71 ± 0.66 ^a^
	11% SRB	0.91 ± 0.08 ^b^	4.64 ± 0.00 ^c^	114.98 ± 0.00 ^b^	15.92 ± 1.25 ^b^	78.14 ± 1.32 ^a^	5.93 ± 0.15 ^ab^
7d	RB	0.52 ± 0.00 ^b^	3.33 ± 0.00 ^a^	100.00 ± 0.00 ^b^	0.06 ± 0.02 ^d^	85.72 ± 0.05 a	14.22 ± 0.64 ^a^
	3%SRB	1.52 ± 0.00 ^a^	9.79 ± 0.81 ^a^	126.45 ± 9.94 ^a^	0.48 ± 0.11 ^c^	91.37 ± 4.33 ^a^	8.15 ± 4.39 ^a^
	5%SRB	0.71 ± 0.07 ^c^	3.04 ± 0.00 ^b^	54.74 ± 4.30 ^c^	18.98 ± 0.45 ^b^	74.88 ± 0.51 ^b^	6.14 ± 0.06 ^a^
	7%SRB	0.72 ± 0.06 ^c^	3.05 ± 0.00 ^c^	133.05 ± 8.52 ^a^	18.79 ± 7.57 ^a^	73.90 ± 38.32 ^c^	7.31 ± 0.85 ^a^
	9%SRB	0.72 ± 0.06 ^c^	3.05 ± 0.00 ^c^	114.96 ± 0.00 ^ab^	17.64 ± 0.06 ^b^	75.39 ± 0.56 ^b^	6.97 ± 0.55 ^a^
	11%SRB	0.72 ± 0.06 ^c^	3.05 ± 0.00 ^c^	132.19 ± 0.00 ^a^	19.34 ± 2.18 ^b^	73.84 ± 2.02 ^b^	6.82 ± 0.62 ^a^

Different letters in the same row indicate significant differences (*p* < 0.05). Data represent the mean (±SD) from three independent replicate experiments. epresents the *Lactptococcus-thermophilus*-fermented rice bread.

## Data Availability

The original contributions presented in the study are included in the article, further inquiries can be directed to the corresponding authors.
